# Network governance forms in healthcare: empirical evidence from two Italian cancer networks

**DOI:** 10.1186/s12913-020-05867-2

**Published:** 2020-11-09

**Authors:** Anna Romiti, Mario Del Vecchio, Gino Sartor

**Affiliations:** 1 Department of Experimental and Clinical Medicine, Health Services Research Unit, University of Florence, Florence, Italy; 2 Department of Experimental and Clinical Medicine, Health Services Research Unit, University of Florence, Florence, Italy; 3 SDA Bocconi, Bocconi University, Milan, Italy; 4 School of Specialization in Hygiene and Preventive Medicine, University of Florence, Florence, Italy

**Keywords:** Governance modes, Healthcare, Networks

## Abstract

**Background:**

This study focuses on the application of Provan and Kenis’ modes of network governance to the specific field of public healthcare networks, extending the framework to an analysis of systems in which networks are involved. Thus, the aim of this study is to analyze and compare the governance of two cancer networks in two Italian regions that underwent system reconfiguration processes due to reforms in the healthcare system.

**Methods:**

A qualitative study of two clinical networks in the Italian healthcare system was conducted. The sample for interviews included representatives of the regional administration (*n* = 4), network coordinators (*n* = 6), and general and clinical directors of health organizations involved in the two networks (*n* = 25). Data were collected using semi-structured interviews.

**Results:**

Our study shows that healthcare system reforms have a limited impact on network governance structures. In fact, strong inertial tendencies characterize networks, especially network administrative organization models (NAO). Networks tend to find their own balance with respect to the trade-offs analyzed using a mix of formal and informal ties. Our study confirms the general validity of Provan and Kenis’ framework and shows how other specific factors and contingencies may affect the possibility that cancer networks find positive equilibria between competing needs of inclusivity and efficiency, internal and external legitimacy, and stability and flexibility. It also shows how networks react to external changes.

**Conclusions:**

Our study shows the importance of considering three factors and contingencies that may affect network effectiveness: a) the importance of looking at network governance modes not in isolation, but in relationship to the governance of regional systems; b) the influence of a specific network’s governance structure on the network’s ability to respond to tensions and to achieve its goals; and c) the need to take into account the role of professionals in network governance.

## Background

### Introduction

Network-based modes of organization have emerged in many public services. In the healthcare sector, they are considered useful tools to define patients’ care pathways and to help knowledge and practice sharing among organizations and professionals [1]. In the case of cancer services, the reconfiguration to network organizational forms has become the norm rather than the exception [2].

Although networks were at first mainly voluntary groups of professionals, they have since become regulated, structured, and institutionalized by governing bodies to pursue central targets and to concentrate healthcare services [1]. These networks, referred to as *mandated networks* [3], can be seen as a completion of the institutional structure of a healthcare system, increasing its complexity by adding a horizontal dimension to the vertical hierarchy.

The governance structure is crucial for network functioning and includes both formal and informal aspects. As pointed out by Iedema et al. [4], the governance of clinical networks has to be studied according to the dynamics and equilibria of healthcare systems. In fact, the creation of networks typically entails reconfiguring healthcare services to a network-based perspective while preserving the autonomy of the participant organizations. However, the reverse effect, that is, the effect of healthcare reconfigurations, such as merger processes, on networks should also be taken into consideration.

Italian regional healthcare systems are interesting cases for understanding and interpreting the position and role of clinical networks in changing healthcare systems. In fact, as every region’s healthcare system has its own structure, different examples of clinical networks have emerged [5]. In this article, we analyze and compare the governance of two cancer networks in two Italian regions that underwent system reconfiguration processes to explore the influence healthcare organizations’ reconfigurations, such as merger processes or the development of new institutional layers (intermediate bodies), have on pre-existing clinical networks.

The literature on network governance has provided indications of governance characteristics that are preferable in certain contexts or that fit better together but has provided little empirical evidence [6–8]. The main and original aspect of this article is the application of the Provan and Kenis model [6] of the governance mode of networks to healthcare networks and its extension to include the impact of reforms on different types of mandated networks. Our research questions are as follows: (a) What are the magnitude and directions of impacts generated by healthcare institutional transformations on two different Italian cancer network governance structures? and (b) Do different network models react differently to system reforms in order to reach a balance between the competing needs identified by Provan and Kenis [6] (efficiency and inclusivity, internal and external legitimacy, and flexibility and stability)?

This paper is structured as follows. First, we review the literature on networks, with a focus on mandated networks and clinical networks. We then define the methodology of our study, followed by the description and comparison of our two cases based on a theoretical framework obtained from the literature on mandated networks. We conclude by summarizing and discussing our key findings.

### Literature

In the public organization domain, Provan and Kenis ([6] p. 236) defined a network as “groups of three or more legally autonomous organizations that work together to achieve not only their own goals but also collective goals.” Organizations join networks to gain legitimacy, enhance their effectiveness, attract resources, and address complex problems [6].

Collaboration may start among network members themselves *(voluntary networks)*, but can also be imposed by a third party, such as an institutional authority *(mandated networks)* [9]. In mandated networks, this third party, often referred to as the regulator, plays a key role in specifying the scope of the network, the financing framework and the distribution of resources and benefits, the eligibility or mandate to participate, the rules for relationships among members, the timing of actions, and the control mechanisms [10, 11]. In highly institutionalized systems, such as public healthcare systems, voluntary networks frequently evolve into mandated networks through institutionalization [12], and these mandated networks often need to achieve the commitment of and legitimacy from members to reach their goals. According to Rodríguez et al. [3], relationships among organizations in highly institutionalized systems are similar to relationships among business units within the same firm. This is true for Italian regional healthcare systems, where the regional authority can be considered a single entity and local health organizations (LHOs) as the business units [13].

The literature on networks has developed many branches (networks’ structural characteristics*,* network formation processes [14], and network effectiveness or performance [15]). One branch treats networks as mechanisms of coordination, often referred to as *network governance* [6]. In this article, we will refer to the latter branch of literature and focus on mandated networks in the public healthcare sector. We will refer to the framework developed by Provan and Kenis [6], who identified three governance models:

- The *shared organization model* where governance is accomplished informally (i.e., in the absence of hierarchy), through the uncoordinated efforts of stakeholders, or formally, through regular meetings of designated organizational representatives.
- The *lead organization (LO) model*, where one of the organizations in the network, chosen by members or mandated, assumes the responsibility for the network’s administration, receives resources from members, or intermediates access to external funds. To set up such a model, one organization needs to have sufficient resources, legitimacy, and/or a central position in the flow of clients/patients to play a leading role [6].
- The *network administrative organization (NAO) model*, which occurs when a single individual (network facilitator or broker) or a formal organization consisting of an executive director, staff, and board, including all or a subset of network members, is set up for network governance and does not provide its own set of services. The administrative organization may be a government entity or a non-profit organization.

These governance models, according to Provan and Kenis [6], are permeated by three essentially contradictory principles: inclusiveness vs. efficiency, internal vs. external legitimacy, and flexibility vs. stability.

First, networks must reach a dynamic balance between efficiency and inclusivity of members. Shared-governance systems, mostly relying on a mode of clan governance to enhance coordination [16], that is, they are organized around shared values, trust, and reputation [3], may be enthusiastic and inclusive, but may lack efficiency, especially if members become numerous and spread out geographically, or they may suffer from *collaborative inertia* [17]. To increase efficiency, networks tend to shift to lead organization models, where direct involvement––but consequently inclusiveness––are significantly reduced. An NAO could balance these tensions, as its board includes members of organizations within a set of formal rules and structures, but may be seen as bureaucratic and less efficient, and members may not feel accountable for network choices [18]. Ideally, network involvement should occur at several hierarchical levels in the organization, thereby gaining the participation, commitment, and engagement of all members (*multiplexity)* [18]. In more complex governance models, such as NAO and lead organization models, interactions may only occur at higher hierarchical levels, reducing the commitment of the base. In NAO models, if all organizations participate in every decision, the network efficiency may suffer from *over-embeddedness* [19].

A second tension between internal and external legitimacy needs to be accounted for.

Internal legitimacy is based on shared values and knowledge, trust, reputation, and goal consensus [20]. This is the result of two different dimensions:

- The commitment of members to the network’s goals can also depend on the competitive patterns among organizations and the potential benefits that members receive from these interactions.
- Clinical networks can have network brokers that play an active role. Some authors [21] noted that the abilities, management style, and leadership [22, 23] of the network manager, also called the facilitator or network broker, are key factors in solving tensions, and building and maintaining commitment (what Agranoff and McGuire [24] call *mobilizing*). To do this in clinical networks, the network broker needs to have a robust endorsement from the professionals [25].

External legitimacy is the value of the network for external stakeholders, such as local or national governments, or eminent public or private bodies.

Shared organization models, which seem suitable for addressing internal legitimacy even though clan mechanisms may result in divisions and distrust, are less easily recognized and legitimated by external stakeholders. On the contrary, lead organizations in particular but also NAOs appear more suited to represent networks externally as unique structures, but they may encounter issues with internal legitimacy.

Third, a balance between flexibility and stability has to be found. Through networks, organizations can work with one another to achieve specific goals that require flexibility in order to share resources, knowledge, and expertise. Hierarchies alone cannot readily accomplish such a degree of flexibility [26]. At the same time, however, a high level of flexibility and adaptability, also typical of shared governance, is likely to be difficult to sustain in terms of legitimacy. Networks must then focus on stability––which can be extended to stability over time (i.e.*,* sustainability and stability of mechanisms)—to maintain legitimacy. The most obvious way is by building a formal hierarchy, although this may destroy the original intent of the network as well as alienate most participants. The regulator could actually over-formalize and constrain roles and relationships at the expense of flexibility [20]. Stability is also related to and influenced by goal consensus on long-term outcomes or process-oriented objectives [27] and by stakeholders’ perceived effectiveness of the network. NAO and lead organization network models tend to be more formalized and stable, and are usually established after networks have started to mature.

Developing a governance structure requires a frequent reassessment of the balance between the aforementioned tensions. How these are managed is critical for network performance [6] and development.

The evolution of the governance structure of a network is important as it influences its dynamics [28]. The structure of the network and its governance may also change as a result of external changes, such as reforms to the systems to which they belong.

According to some authors [29], who use, among others, the framework of Provan and Kenis [6], the literature on the dynamics of network structures in response to external changes can be divided in two bodies of research: (a) emergent networks and (b) orchestrated networks. In the case of public networks, emergent networks can be assigned to the shared models, while orchestrated networks can be referred to lead and NAO models [6].

In emergent networks, structural change is the “unintentional” [30] result of a “myriad of intentional actions carried out by network organizations in the pursuit of their individual goals” ([29] p. 354).

In orchestrated networks, governance is the result of the intentional action of a lead organization at different stages of the network’s lifecycle. In more mature stages, the structural dynamics are characterized by inertial tendencies. To respond to external changes, lead organizations tend to use informal network coordination mechanisms.

Referring to networks embedded in public systems, some authors [31] showed that reforms had a limited impact and, moreover, networks needed a long to adjust. Inertial tendencies featuring structure dynamics in a network are a recurring theme in network research [30].

Based on this reasoning, we expect that, as in other healthcare public networks, health reform policies should have a limited impact on network organizational structures and that inertial tendencies, which characterize structure network dynamics, are strongest in the mature stages of networks. Moreover, in the lead organization form, the responsivity of the network to external changes can be greater than in the NAO form because the lead organization plays a specific role in the network [32, 33]. Despite the limited impact of reforms, each network is able to find its own balance with respect to the trade-offs analyzed, using a balanced mix of formal and informal network ties.

## Methods

### Research design

This study assessed the different governance structures of two clinical networks in two Italian regions. This research is based on case studies that are particularly appropriate for investigating healthcare systems’ complexity [34, 35].

### Case sampling

The sampling strategy was purposive [36]. We selected cases from Italian cancer networks that are mandated by the regional authority and considered the most consolidated [37], while excluding those that have not experienced a major system reconfiguration [38]. As in others researchers’ research, a comparison between two cancer care networks was considered appropriate [39].

### Data collection

First, we collected information on national and regional regulations concerning clinical networks. A preliminary interview protocol was developed and adapted for LHO members (general director, clinical director, oncology department directors, and oncology unit directors) and regional healthcare office directors, both chosen according to the theoretical framework and our research questions. The protocol was refined after conducting the first interviews. The interview protocol is shown in Appendix. The aim of the interview protocol was to identify the three trade-offs (efficiency vs. inclusiveness, internal vs. external legitimacy, flexibility vs. stability) described in the literature, differences in trade-offs between the two regions, and how institutional change impacted the three dimensions.

Interviewed subjects are described in Table 1, according to the organization they belong to, their role, and time in the current position. We interviewed 28 men and 8 women.

**Table 1:** Interviewed subjects, their organization roles, and time in the current position

		Number and mean time in charge (years)	
Organization	Role	Veneto	Tuscany
***Region***	*Regional Healthcare Offices Director*	3 (8.2)	1 (0.1)
***Network Coordination structures***	*ONC/IOSPN*^a^ *member*	1^b^ (4.7)	5 (4.5)
***LHO/UHO***	*General Director*	6 (2.8)	3 (5.4)
	*Clinical Director*	4 (4.1)	1 (1.1)
	*Oncology Department Director*	2 (2.6)	3 (1.9)
	*Oncology Unit Director*	4 (1.8)	3 (6.8)
***Total number of interviews***		**20 (4.0)**	**16 (4.4)**

^a^*ONC* Oncologic network coordination, *IOSPN* Institute for Oncologic Studies, Prevention, and Networks; ^b^Other ONC members are regional healthcare office directors and thus listed above

The interviews were conducted by three researchers (a medical doctor intern in hygiene and preventive medicine, and two scholars in the management field) from September 2018 to July 2019. Interviews lasted 30–60 min, and were recorded and transcribed for data analysis. According to Leavy ([40] p. 295), a combination of the two broad interviewer styles (receptive and assertive) should be used as they “fertilized each other in a productive ways”, on the basis of the different roles of the interviewees and the progress of information collected.

### Data analysis

**Fig. 1:**
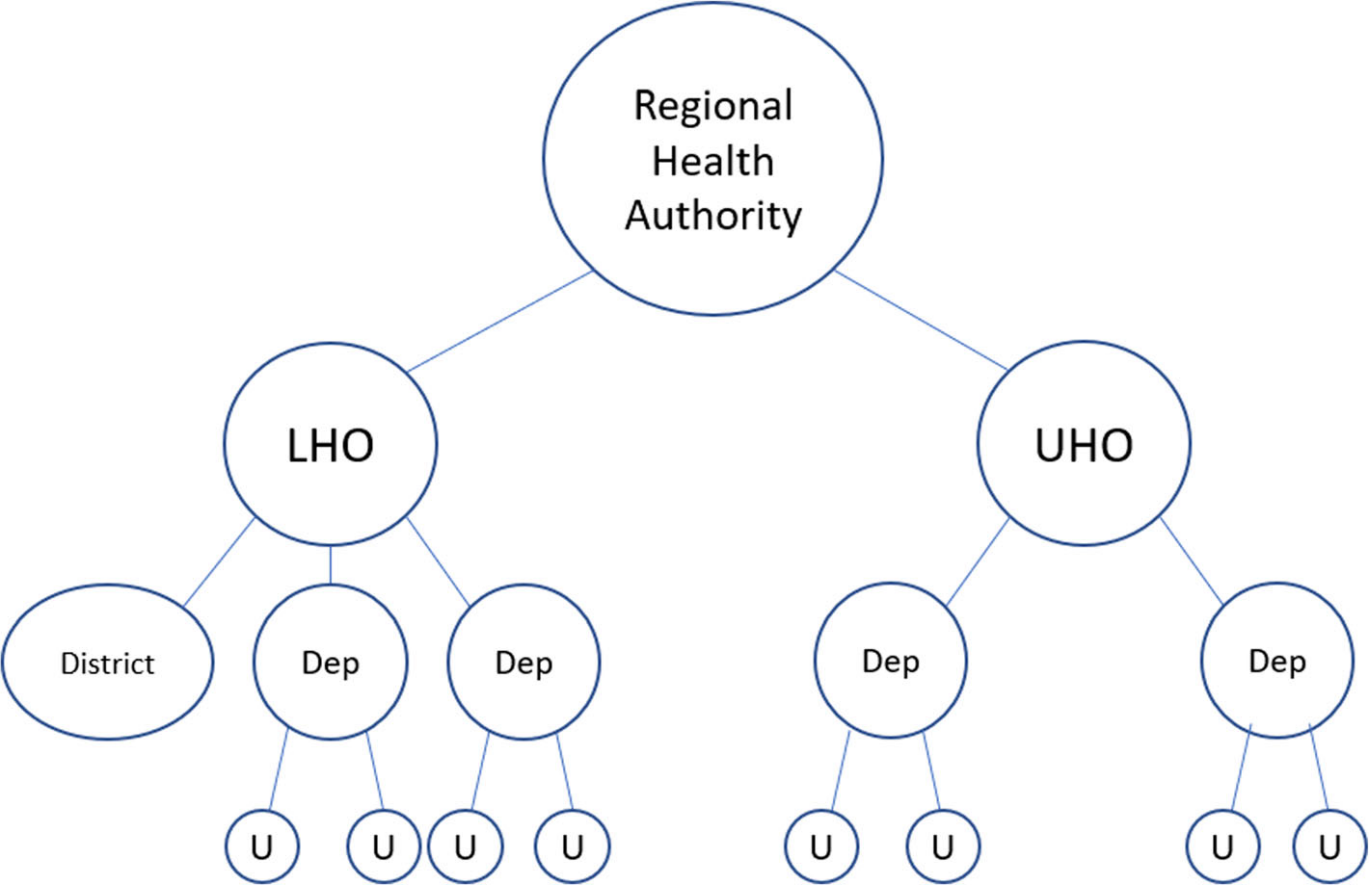
Basic structure of the regional healthcare systems. LHO: Local health organization; UHO: University hospital organization; Dep: Functional Department (e.g., Oncological Department); District: part of the LHO responsible for territorial (extra-hospital) services; U: Clinical unit

**Fig. 2:**
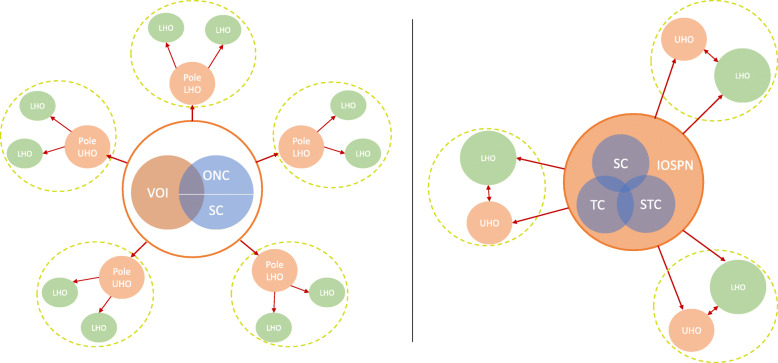
Veneto (left) and Tuscany (right) network structure. VOI = Veneto Oncological Institute; ONC = Oncological network coordination; SC = Scientific committee; LHO = Local health organization; UHO = University hospital organization; IOSPN = Institute for Oncologic Studies, Prevention, and Networks; TC = Technical committee; STC = Strategic committee

One of the authors coded the interview transcripts using descriptive coding, summarizing the main topics of significant passages in the transcripts, and used the constant comparative method to compare the answers of interviewees with the same/different roles and working in the same/different organizations and regions [41]. We then exported codes and significant quotations to a synoptic table from which researchers identified key emerging themes and concepts related to the three investigated dimensions and their evolution after healthcare system reforms. We integrated the results of the interviews with our document analysis to assess the consistency or discrepancy between official documents and statements in interview transcripts. As suggested by Lambotte and Meunier [42], the qualitative research brings together interviews, observations, archival documents (for instance, published clinical pathways), images, and text, that they called “the patches”, and this makes it possible to create a quilt from broad and varied sources and to connect the many parts to make the whole [43]. Integrating narratives and images, as suggested by Jennings et al. [44], allowed the researchers to better understand the design and functioning of the two networks selected for this paper (see Figs. 1 and 2).

Based on the literature [45], we presented multiple sources of evidence as a basis for trustworthiness and credibility. In particular, trust stems from co-construction and interpersonal contacts with participants [46]. In this paper, the different sources are the perceptions of interviewees that had the same role or different roles inside the two networks (clinical role, institutional role). Furthermore, this research’s trustworthiness and credibility are also assured by the fact that the questions that were asked in the interviews concerned a judgment of the network and not the activities carried out by each interviewee. This mitigated the risk of intentional bias in the answers [47].

Preliminary results for the first case were presented at a congress on clinical networks, while preliminary results for the second case were discussed in a meeting with one of the main governance boards of the network.

### Analysis of cases

The two regional healthcare systems and networks we studied were mainly public, both in terms of financing and the provision of services. Main actors in the systems are Local Health Organizations (LHOs) and University Hospital Organizations are UHOs. LHOs receive regional funding and either provide healthcare services directly or buy services from UHOs or private accredited providers using predefined prices (e.g., diagnosis-related groups). Both LHOs and UHOs, whose CEOs (general directors) are appointed by the regional authority, are internally organized into functional departments according to disciplines (e.g., the oncology department), which are in turn divided into units according to structural location. Oncology department directors are appointed by the general director of each LHO/UHO. LHOs also provide primary care on a district level, where some primary services are directly provided (e.g., blood samples and home nursing services), while other services are provided by private agents, such as general practitioners, who are responsible for ensuring citizens’ access to healthcare and are mainly paid on a per capita basis.

The basic structure of the healthcare systems is summarized in Fig. 1.

In the last decade, this basic architecture has undergone major institutional changes in many regions, including a) the creation of intermediate bodies between the region and the LHOs as a part of the overall regional governance structure, and b) a decrease in the number of LHOs and an enlargement of their area [48]. This process of mergers and centralization has been accelerated by national laws defining minimum volumes of activities for healthcare structures and the imposition of centrally monitored targets. Another important trend in Italian healthcare systems, recently enhanced by national laws, is the development and institutionalization of clinical networks, which are the focus of our paper.

LHO merger processes have been more intense in Tuscany, resulting in three LHOs servicing an average population of more than 1 million inhabitants, twice as much as in Veneto. In addition, in terms of geographical extension, LHOs are almost four times larger in Tuscany than in Veneto. Every LHO in Tuscany has at least a respective UHO in its territory, while only two LHOs in Veneto have a UHO. These and other differences between the regions are shown in Table 2.

**Table 2:** Regional population and healthcare system characteristics (2018)

	Veneto	Tuscany
***Population***	4,903,722	3,736,968
***Area (km***^***2***^***)***	18,345	22,987
***Population Density (inhabitants/km***^***2***^***)***	267	163
***Number of LHOs (after merger)***	9	3
***Number of LHOs (before merger)***	21	12
***Extent of LHO number reduction after merger***	− 57%	− 75%
***Number of UHOs (after merger)***	3^a^	4^b^
***Average LHO population (after merger)***	544,858	1,245,656
***Average LHO area (km***^***2***^***) (after merger)***	2038	7662

^a^A UHO is specific for cancer care (Veneto Oncological Institute, VOI)

^b^ A UHO is specific for pediatric care

In terms of the structure of providers, the network in Veneto has a multilevel *hub and spoke* structure, with a clear tendency to centralize complex cases into hospital hubs (called *poles*) and, in cases requiring higher levels of care to the two UHOs and to the Veneto Oncological Institute (VOI). The VOI is an oncological-only UHO that can be considered the lead organization of the network. In Veneto, LHO merger processes have not progressed to the point where the traditional hierarchical structures of providers (LHO peripheral hospitals, LHO provincial hospitals, and UHOs) have been discarded. Instead, the merger process in Veneto made the LHOs less ambiguously connected to a specific hub. The involvement of the “spokes” in the network as not only “patient-senders” should be guaranteed by their participation in the decision-making process and by the possibility of becoming regional reference centers for specific pathology and procedures.

The structure of the network in Tuscany has a less clear hierarchy of providers in terms of the volume of activity or leadership roles. In fact, extensive merger processes (from 12 to 3 LHOs) made the new LHOs bigger and more influential compared to UHOs, somehow dismantling the traditional hierarchy of providers and determining more complex patterns of centralization with no explicit reference to a *hub and spoke* model in the network statutory law.

Primary care providers, both in Tuscany and Veneto, are responsible for the initial cancer diagnostic phase (unless there is a screening program), for the later follow-up treatment, and for the treatment of other health issues which may pre-exist or emerge after the cancer diagnosis. Palliative care and hospice care are also included in primary care services.

In terms of governance structure, the two networks are characterized by the existence of an organization at the head of the network. However, considering the governance models proposed by Provan and Kenis [6], the Veneto network is a form of lead organization network, although it shows some hybrid characteristics of an NAO model. The Tuscany network is almost purely a NAO model.

Network governance in Veneto is based on two levels: a lower level of coordination consisting of five *pole* commissions, each of which includes some LHOs and can include an UHO. The presence of two coordination levels enhances *multiplexity* [18], ensuring a broader participation and commitment of all hierarchical levels of member organizations. The demands and proposals of professionals are discussed, both formally (in *pole* commissions or within the scientific committee) and informally (directly with the coordinator, a clinician designated by the regional healthcare authority), and then gathered and summarized by the network coordinator, who then brings the synthesis to the upper level of coordination (ONC) in which regional offices and members of the intermediate body participate.

The ONC validates care pathways and specifies criteria to identify regional centers of reference for every type of cancer and for drug prescriptions, coordinates and monitors the activity and the organization of network nodes and network commissions, and coordinates clinical research. The ONC is supported by a scientific committee, composed of LHO and UHO representatives, general practitioners, and patient associations. The VOI may be seen as the lead organization of the network, according to the Provan and Kenis model [6]. In fact, four elements show the strong leading role of the VOI in the Veneto network:

- The ONC physically occurs at the VOI.
- The VOI is the biggest oncological provider in the network.
- The VOI receives funding for network-specific activities (e.g., clinical research coordination or network website development).
- The network coordinator in charge––at least at the time of our research––is the director of an oncology unit at the VOI.

The fact that the ONC is formally independent from the VOI indicates an attempt to evolve to a hybrid NAO-LO form of network governance.

Network governance in Tuscany is based on a single formal level of coordination that takes place in an institute, the Institute for Oncologic Studies, Prevention, and Networks (IOSPN), whose clinical activities are minimal (oncological screening for the city of Florence). Thus, it is mainly devoted to network coordination for which it receives regional funding. The coordination is achieved by three committees chaired by the IOSPN general director:

- A strategic committee, in which regional office members and the general directors of all LHOs and UHOs participate; it is responsible for planning and monitoring network activities, through a plurennial strategic document.
- A technical committee, in which directors of oncology departments and regional offices members participate; they are responsible for supporting the strategic committee in the definition and implementation of activities planned by the strategic committee and expressing opinions on the plurennial document.
- A scientific committee, whose members are designated partly by the strategic committee and partly by the IOSPN general director; they are responsible for supporting other committees and expressing opinions on the plurennial document.

In Tuscany, the presence of a single formal coordination level means that most of the coordination efforts are now in charge of oncological departments inside LHOs and UHOs (Table 3).

**Table 3:** Differences between the networks in terms of structure and governance

	Veneto	Tuscany
*Providers structure*	Multilevel (*hub and spoke)*	Horizontal
*Governance model*	Lead Organization	Network Administrative Organization
*Coordination levels*	2	1
*Higher-level coordination committees*	2^a^	3^b^
*Lower-level coordination committees*	5^c^	–
*Year of network implementation*	2014	2001

^a^*ONC* Oncologic network coordination and scientific committee; ^b^Strategic committee, technical committee, and scientific committee; ^c^Pole commissions

## Results

Results will be presented according to Provan and Kenis’ [6] network contradictory logic. For each contradictory logic, we assessed the impact of the healthcare system reconfiguration.

### Efficiency vs. inclusiveness

As mentioned above, in a network that increases its efficiency, inclusiveness can be significantly reduced. Furthermore, in the specific case of health organizations, inclusiveness has two dimensions: professional and institutional inclusiveness.

In Veneto, the recent LHO merger and creation of intermediate bodies had a positive direct impact on institutional inclusiveness. Before the merger, smaller LHOs tended to be excluded in the decision-making processes. After the merger, the creation of mostly provincial-level LHOs, each with a clearly defined hub hospital, made the network structure clearer and enhanced institutional inclusiveness by clearly identifying the role of each node. One LHO general director explained: *“Now spokes feel free to send patients to hubs without feeling belittled, whereas they may feel it as a ‘patient leak’ even if they would have treated them inappropriately.”* The merger also reflected on professional inclusiveness: “*There is a certain satisfaction for a professional, even if located in a spoke, to implement care paths that are used and recognized all over the world.*” The shift in administrative and management functions from the network to an intermediate body (Azienda Zero in the Veneto case) had a positive effect of increasing the efficiency of the network by unburdening it from tasks important but peripheral to the mission, such as building a unique regional electronic medical record or analyzing administrative data. On the other hand, an initial sense of expropriation or reduced inclusiveness may have occurred: “*I spent a lot of time preparing the electronic medical record. The network was somewhat surpassed by the region in this.*”

The central role played by the coordinator, both in terms of inclusiveness and efficiency, had not changed after healthcare system reforms. A regional office director remarked: “*In taking decisions, I have the support of the coordinator who has already discussed the topic with network representatives*” and professionals. An oncology department director noted: “*The coordinator is a sort of prime minister, supported by the scientific committee, who are the ministers. The prime minister is not just an institutional role; he decides whether a choice is good or not. He has expertise and charisma. We need a leader because you cannot just listen to everybody without synthesis*.”

The inclusive structure of the Tuscan NAO model has not formally changed after the merger processes. Nonetheless, the reform extensively reduced the number of LHOs and the need for lower-level coordination, thus simplifying the decision-making process. Participation in network committees is open to all member organizations and, according to an IOSPN director, this guarantees institutional inclusiveness: *“Having only three large LHOs and an NAO makes the network easier to manage and gives it more potential. With many LHOs the process was more chaotic.”* However, broad inclusiveness, especially at higher coordination levels, may also have disadvantages in terms of decision-making processes and efficiency. This could become too rigid as claimed by a respondent: *“Its weakness is that network participants may rely on the governance entity too heavily. The latter may adopt decision-making processes that seem overly bureaucratic.”* The same opinion is shared by a clinical director: “*There is no conflict between the network and health departments, but the network processes are too long.*”

### Internal vs. external legitimacy

Internal legitimacy depends on the degree of competition among participating organizations and the role played by facilitating mechanisms, such as the role of network brokers or the functioning of the representative system. The two networks show a different pattern of competition among organizations that influences the relationships among members and affects their commitment to the network.

In Veneto, the hierarchical formalization of the hub-and-spoke structure requires a high degree of consensus among both professionals and the strategic apexes of participating organizations. The LHOs merger did not alter competition patterns among LHOs, UHOs, and the VOI. These institutions maintain distinct roles in the network. Most of the competition is played out among UHOs to attract patients from regional and extra-regional LHOs for both financial reasons and prestige. The same hierarchical structure applies to the field of clinical research. In the case of the latter, the VOI has the mandate to coordinate clinical research and keep databases of studies being conducted in the region. This role of the VOI is favorably considered by network members as underlined by an LHO oncology unit director: *“Centralization added real value, especially because the resources were scarce.”*

In Tuscany, the merger distributed more evenly the “market power” among participant organizations, increasing the degree of competition. A network coordinator affirmed: *“After merger, larger LHOs started competing with UHOs.”* The increase in the level of competition made it more difficult to exploit potential synergies in the system. “*Before the merger, groups of smaller LHOs and their respective UHOs had regular meetings, which are now occurring more sporadically. We were more accustomed to working together as professionals, to hold scientific or clinical meetings, we used to have a research coordination office that is no longer there*,” as affirmed by a technical committee member and LHO oncology unit director.

Most of the competition is perceived to operate between an LHO and its respective UHO, and a reduced LHO–UHO coordination capacity is seen as a drawback of the merger processes, as noted by a LHO oncology unit director: *“There’s still this misunderstanding that only UHOs should do high-level activities particularly in the field of hospital care. In Tuscany, this is no longer the case, since we have LHOs that can compare with UHOs in terms of competencies. This can create conflict between LHOs and UHOs.”* However, the network coordination committees, where LHO and UHO department directors and general directors regularly meet, are considered the right places where these kinds of conflict can be resolved, as two different respondents declared. As an UHO clinical director noted: *“We had LHO–UHO formal contacts before mergers on many topics. Now I perceive wider divergences between LHOs and UHOs, but they may well be overcome by the coordination efforts of the network.”* An LHO oncology department director said: *“LHOs now have a peer-to-peer dialogue with UHOs and we perceive some relationship difficulties, for example, on the issue of breast cancer units. These conflicts should be resolved in the network committees.”*

The second aspect of internal legitimacy refers to the network broker and the network committee’s roles. In both regions, the attitude, leadership, and clinical authority of the coordinator in office are recognized as important elements of the network.

In Veneto, the role of the network broker did not change substantially after the reforms and remained highly legitimated and perceived as “belonging” to the professional rather than to the regional/political component (the coordinator of the network is an oncology unit director). As noted by an LHO oncology unit director: *“The coordinator is assertive, engaging; he has given us stimuli to change our mentality.”* On the other hand, some internal legitimacy issues have emerged because of the central role of the VOI; some participants see the network as a means for the VOI to pursue its own goals. The new role of Azienda Zero in the ONC might be seen as an attempt to balance the power of the VOI in the network.

In Tuscany, the network broker and the IOSPN are perceived by professionals as regional components of the network. This has become more evident after the merger of LHOs and the lengthening of the chain of command between professionals on one side and directors of departments and LHO directors on the other.

External legitimacy refers to how the network is perceived by external stakeholders, such as funders, regulators, the public, and the media. We will mainly refer to the perception of funder-regulators, which coincides with the regional authority in the analyzed networks.

In Veneto, the leading role of the VOI remains crucial because it physically represents the network center. The ONC is a useful tool for regional authorities, and provides a unique interface with the broker. As one interviewee remarked: *“The strong leadership of the coordinator on clinicians played a crucial role since it offered a solid ground for clinically based decisions and helped in the implementation processes.”* After the reforms, while the lower level structure of the network has been reorganized by the LHO merger, in the upper level (ONC) members of the intermediate body were introduced. Some tasks shifted from the network and the region to Azienda Zero. However, final decisions are still taken at the regional level, as noted by a representative of the regional authority: “*If the proposal coming from the network through the coordinator is not convincing, we decide differently.”* The decision to create a link between the network and Azienda Zero is also an attempt to include the network in a “network of networks”. The intermediate body has the ambition of becoming the coordinator of all regional clinical networks. In fact, patients do not “belong” to a single network. For example, a patient may be diagnosed with both diabetes and cancer, and thus should be tracked by two networks in an integrated manner. In Tuscany, not only is the network administrative organization (IOSPN) legitimated by the regional authority, it is also as part of the regional authority and decisions are taken in its committees. With the concentration of network governance in the IOSPN after the LHO merger and network reform, the link between member organizations and regional authorities has become closer. In addition, the network broker of the network recognizes his role as a liaison between participant organizations and the region, as he himself states: *“Unlike what happens in other networks, in Tuscany there is a strong need for a liaison figure with the regional government and administration. I have a mandate from the regional government. In other regions, the network is more horizontal. In Tuscany the network has a professional base but it is strongly institutionalized.”*

### Flexibility vs. stability

For public healthcare networks, changes in the environment and the consequent need for flexibility may have different sources. Considering their impact and frequency, system reforms are among the most important. In this instance, it is the regional administration that, through top-down decision-making, changes the relevant environment in which the network operates and potentially asks for changes in the functioning and governance of the network itself.

The processes of institutional change (mainly the reduction in the number of LHOs) that affected both systems had different levels of intensity and progressiveness. The reform in Veneto was less radical and more gradual compared to what happened in Tuscany where, through an institutional shock, the whole system was completely revised. Consequently, the need for change in the two networks was different. Although at different levels, both would have required a “reassessment of structural mechanisms and procedures in light of new developments, and a willingness to make needed changes” ([6] p. 245). Instead, both systems introduced adjustments involving more informal mechanisms than formal procedures and rules (the structure). However, some differences can be found.

In Veneto, a lower degree of formalization of the network, coherent with the LO form of governance, made it possible to absorb the impact of external changes without any detectable modification to the network’s structure. Pressure from the system for better representation of the network in the regional logics has elicited intense dialogue among the involved actors. As noted by a member of a regional office: “*A diabetic patients with cancer might or should be taken care of by many healthcare networks (i.e., a diabetology and cancer networks). The regional authority or Azienda Zero should invite networks to dialogue, to avoid issues for the patients.”* According to the literature, this flexibility can be explained in part by the specific role of a lead organization (opposite to NAO forms) in determining the responsivity of the network against external change and the use of informal mechanisms*.*

Tuscany, whose network is older, has gradually strengthened the formal structure of the network to improve its sustainability. This can be seen both as a sign of network maturity, but also as network evolution towards more hierarchical structures. Owing to its greater degree of formalization, the system would have been in greater need of change in its component parts. The system instead replicated the same governance structure despite major changes introduced by the reform: the size of the organization’s network members was greatly increased and, as a result, their weighting increased noticeably, thereby changing the balance of the power in the network. This is well recognized by a director of an oncology department: *“With the mergers that have created much larger organizations, the network lost strength, it should be rethought in its functions and mission and the structures and mechanisms governance should be redesigned.”* (Table 4).

**Table 4:** Differences between Veneto and Tuscany Model about Provan & Kenis Trade offs

Trade-off	Veneto Model	Tuscany Model
Efficiency vs. Inclusiveness	The new provincial-level LHOs have enhanced institutional inclusiveness through a better definition of the network structure and a better identification of the role played by each node. The shift of some functions from the network to Azienda Zero has had the positive effect of increasing the efficiency of the network by unburdening it from non-mission related tasks, even though an initial sense of expropriation may have occurred.	The merger reform extensively reduced the number of LHOs and the need for lower level coordination, simplifying the decision-making process. However, broad inclusiveness has disadvantages in terms of decision-making processes and efficiency because of the risks of bureaucratization. The coordination of nodes inside new sub-regional LHOs previously under the network responsibility passed on to the LHOs (network of networks).
Internal vs. External Legitimacy	The LHOs’ merger had not altered previous competition patterns among nodes, which maintain distinct roles in the network. Most of the competition is still played out among UHOs. The roles of the lead organization and the network broker have not changed after healthcare system reforms and remain fundamental for the network’s internal and external legitimacy.	The merger determined a more homogeneous distribution of power among participant organizations and increased the level of competition. Most of the competition is perceived to operate between an LHO and its respective UHO. The role of the network broker tends to be identified with regional authority. This has become more evident after the merger of LHOs and the lengthening of the chain of command.
Flexibility vs. Stability	A lower degree of formalization of the network, coherent with the LO form of governance, made it possible to absorb the impact of external changes without any detectable modification to the network’s structure. The flexibility of an LO can be in part explained by the specific role of a lead organization (opposite to NAO forms) in determining the responsivity of the network against external change and the use of informal mechanisms.	Tuscany has gradually strengthened the formal structure of the network to improve its sustainability. The system replicated the same governance structure despite increased size and weight of the members, changing the balance of the power in the network.

## Discussion

The analysis of the two cases provides insight into the functioning of mandated public health networks. We focused on cancer networks for many reasons. From the Italian perspective, cancer networks are the older and more institutionalized forms of clinical networks [49] and serve as references for other networks. They tend, more than other networks, to behave as a system in the general healthcare system. This is also because tumors are considered, especially by patients, as a prevalent condition, thus oncological structures are often seen as and try to be a global provider of services for all the needs of the oncological patient. In synthesis, cancer networks are good examples of very complex networks playing a critical role in the functioning of public healthcare systems. After analyzing modes of network governance, Provan and Kenis [6] proposed three different forms or modes. While there is no single best form, each one is likely to ensure different degrees of effectiveness in relation to certain critical contingency components. More importantly, each form exhibits distinctive advantages and disadvantages in managing three typical tensions that inevitably arise in network functioning and in their response to system changes. Our study confirms the general validity of the Provan and Kenis framework and shows how other specific factors and contingencies may affect the possibility of the two cancer networks finding positive equilibria between competing needs.

### Efficiency and inclusiveness

Inclusive decision-making is a condition for building trust and promoting collaboration among participants, but it is time-consuming and can threaten administrative efficiency in network governance. The institutional framework characterizing lead organization models responds better to efficiency needs, while opening potential gaps in inclusiveness dimensions.

The Veneto case shows two different ways of coping with such gaps. As far as “professional inclusiveness” is concerned, the average dimension of the participant organizations, even after the reorganization, and their mutual dependency facilitate the informal interactions among professionals of different organizations. The network coordinator participates in this intense dialogue and, given his formal role, easily brings concerns and critical issues coming from professionals to the decision-making level. In the case of mandated networks working in structured healthcare systems, organizational inclusiveness should be considered from a wider institutional perspective. LHOs, UHOs, and the VOI itself are all public organizations whose owners are the regional governments that govern the system as a whole through regional administration. The governance form of the network is thus part of a wider governance structure of the regional healthcare system that, in turn, exhibits its own equilibrium between efficiency and inclusiveness. In Veneto, where formal inclusiveness is lower, but the level of trust and collaboration among public organizations and thus the informal inclusiveness is very high, the cohesiveness of the overall system removes some of the pressure for inclusiveness inside the network.

The NAO is a compromise form embodying structured and representative participation in a focused administrative machine; not surprisingly, the IOSPN, as an NAO, must face problems that differ from those experienced by a lead organization like the VOI. In the professional dimension, the representative structure of IOSPN may try to assure the involvement of professionals as individuals, but may experience significant difficulties with genuine networks. In fact the new dimension of LHOs and the persistence of dynamics linked to the previous LHOs, make the Tuscany network a “network of LHO networks” in which “the functioning of each of the internal networks directly influences the efficiency and efficacy of the external network” [50]. From this perspective, the interactions between internal networks and the external regional network are relevant, but they cannot be governed through formal representative mechanisms and what actually happens inside each organization remains a problem for the IOSPN. From an organizational point of view, the limited number of participants, the scarcity of their operative interactions, and the prevalence of dyadic relationships (LHOs and UHOs in the same area) push the IOSPN toward a role of global planning and regulation in which it is not easily distinguishable from the regional administration with its routine administrative burdens. Certainly, the focused mission, technical capabilities, and structured participation of the participant organizations in decision-making are unique characteristics and specific advantages of the NAO form. In any case, such differential advantages over a classical form of coordination through regional administration must be compared to the cost of the necessary coordination between the NAO and the regional government itself. In the Tuscany case, such costs are negligible, given, on the one hand, a long history of personal and institutional cooperation between IOSPN and the region, and on the other hand, the strength and stability of the political leadership.

### Internal and external legitimacy

In both cases, a critical role in managing legitimacy tension is played by a professional figure who acts as the network broker. However, the nature of the role played in practice and the characteristics of individuals appear to be different.

The lead organization form is especially equipped to respond to the challenges of external legitimacy, exploiting the intrinsic legitimacy of the lead organization, which extends its influence to the network as a whole [6]. In Veneto, the external legitimacy of the lead organization (VOI) is visibly reinforced by the role of the network coordinator. He is a well-known clinical leader with a high level of personal legitimacy that extends from the professional (internal) to the institutional (external) sphere [23]. In this case, not only is there no tension between the two sides, but they reinforce each other. Internal legitimacy is a fundamental basis for external legitimacy and, at the same time, the possibility and ability to negotiate for the network with external stakeholders, the region *in primis*, makes the role more legitimate and stronger. The equilibrium in the network between the two competing needs is thus reached by the joint contribution of the governance mode (the presence of a lead organization) and the action of a professional leader playing a formal as well a substantial role. From this point of view, the governance structure of the lead organization itself becomes part of a more general governance mode of the network, which in turn is part of the governance of the regional healthcare system. In fact, the VOI has a general director and a scientific director, but the network coordinator is the director of an important department and plays a substantial role in the actual governance of the institute. In this way, the needs and points of views of the network can have a strong representation in the choices of the lead organization.

The NAO should represent a form able to reach a balance between internal and external legitimacy needs. However, the IOSPN case shows that even when the governance structure is potentially more able to absorb competing tensions, the importance of a network broker from the professional environment cannot be undervalued. This is especially true in a situation where competitive pressures may be particularly strong (UHOs and LHOs insisting on the same catchment area) and the new dimension of participant organization risks make the representative structure of NAO too removed from the actual dynamics of front-line professionals.

Moreover, larger organizations, being more self-sufficient, may have fewer incentives to cooperate and enjoy a stronger external legitimation as single organizations; thus, the centralized administration of the NAO may not be sufficient to represent the network externally.

In this complex situation, governing and managing the IOSPN “machine” is not enough. The personal legitimacy of the IOSPN general director plays a crucial role in keeping together these diverging exigencies. He comes from the professional field and is still recognized by his former colleagues. At the same time, he is very familiar with it and is well known in the administrative, political, and institutional environment of the region. Even in the Tuscany case, the network’s actual functioning and its outcomes are thus the results of the governance form and of the action of a credible leader capable of representing the network at different levels, and acting in different dimensions.

### Flexibility and stability

The last tension that networks have to face is a trade-off between the need for flexibility and the need for stability. Here, the NAO and lead organization forms, even if different, are both positioned on the “stability” end of the spectrum. This is especially true for mandated networks operating inside public healthcare systems, where such networks are part of the institutional framework linking regional governments with public organizations. In Veneto and Tuscany, the VOI and IOSPN play a definitive role in regional governance. Both have the mission to increase levels of collaboration among organizations already coordinated through the institutional hierarchy. In being part of the institutional framework, mandated networks should accompany and be part of the re-adjustment to the system to which they pertain. In line with the original characteristics of networks, as opposed to those of hierarchies and institutions, we expected mandated networks to be among the most dynamic components of the institutional framework of regional systems. However, paradoxically, the IOSPN and VOI had not significantly changed their formal governance structure even on the occasion of what could be considered a major change in both healthcare systems, namely, the reduction in the number of entities to be coordinated and a definite push toward centralization.

The stability of governance forms does not imply that the network did not react in some way to the change in the environment. They marginally readjusted their role in the system while moving in two different directions. The Tuscany network moved toward a more institutionalized role, similar to that of a specialized department of the regional government, now coordinating a smaller number of larger organizations. The Veneto network instead focused its action on the orientation of professional behaviors, redefining its relationships with the regional government and Azienda Zero in particular.

It is important to underline that the change in network functioning did not appear to be the result of an intentional design, nor did it touch the formal structures of both networks. The persistence (stability) of governance forms may have different sources and explanations. Looking at the Veneto and Tuscany experiences, we propose three different complementary and interpretive perspectives.

The first refers to the role of political rationality and its limitations in the process of change in political and institutional systems. In both cases we analyzed, the reduction in the number of public institutions was the result of external (political) pressure, the need to change something, rather than due to internal (functional) exigencies of the systems. This change has probably focused on the more visible and structural part of the systems, leaving aside networks perceived as mechanisms to be adjusted rather than structures to be formally modified.

The second perspective considers the importance of the formal structures of the VOI and IOSPN in relation to their actual role in the network’s functioning. Flexibility and adjustments may well occur even within an unmodified set of formal rules (the network governance forms), considering that a relevant component of the coordinating function rests on the capacity to exert influence outside a hierarchical framework. This means that perceptions, behaviors, and individuals are more important than formal rules. From this point of view, future research could explore the conditions for a positive coexistence of formal and informal mechanisms as well as the individual characteristics of the broker.

The third perspective looks at mandated networks as a part of a regional governance structure aimed at governing and controlling public entities in charge of providing services to the population. In terms of system governance needs, the reduction in the number of LHOs has not been perceived as a major change in the nature of the system and has not triggered any actual reassessment and modification of governance structures and mechanisms. Notwithstanding all the proposed explanations, from a more dynamic point of view, the relative insulation of such mandated network governance structures from changes in the institutional environment, of which they are an important component, remains to be fully described and understood.

Two lines of research could be explored further in the future. The first one looks at mandated networks from the specific perspective of the governance of healthcare systems, while distinguishing between structures (institutions) and mechanisms. Mandated networks are part of both worlds and may well initially be considered part of institutional design when they are established and later be seen just as mechanisms to be adjusted only if and when the need arises. The second focuses on the professional nature of the networks considered. If networks are seen as specialized forms of governance, mainly devoted to influence professionals’ behaviors, their connections with the overall institutional architecture of the system weaken and their dynamics may follow independent and different paths.

## Conclusions

Using the framework proposed by Provan and Kenis [6], we analyzed the case of two regional cancer networks in the context of the Italian national healthcare system. The selected networks share two characteristics that make them interesting and must be taken into consideration when interpreting the results. The first is the professional nature of activities to be coordinated that influences in many respects the ways in which individuals and organizations can be oriented. The second feature is their institutional collocation. Even if they involve organizations that enjoy large degrees of autonomy, they are part of a unitary public system that uses them as a component of a definite regional governance architecture.

In comparison with previous studies that examined “generic” networks, our analysis allows specific traits of this kind of network to emerge, opening up fresh perspectives for future studies. Our results can be used for other networks, especially for networks devoted to chronic conditions as opposed to acute-care networks (e.g., diabetes).

The role that professionals play and the power that they can exert in the functioning of healthcare systems and organizations are well-known issues in managerial literature. From the specific perspective of networks, significant attention has been devoted to clinical networks and professional behavior. Our research confirmed the importance of taking into account the role of professionals, distinguishing in the analysis the professionals from the organizational side. In the healthcare context, networks are complex mechanisms in which the coordination of organizations cannot guarantee a coherent alignment of individual behavior. Formal arrangements and procedures, typical of structured governance modes, may cope better with organizations, but they must be complemented with adequate informal mechanisms and other conditions if they want to influence professionals. The cases we analyzed highlighted the critical role of network brokers with a strong professional background and legitimacy in keeping together the professional with the organizational dimensions of network functioning.

The collocation of the objects of this analysis into a wider institutional framework suggests the need to look at networks’ governance modes not in isolation, but in a tight relationship with the governance of regional systems. From this perspective, some of the “classic tensions” may be alleviated by the characteristics of regional governance, as in the case of legitimacy or inclusiveness offered to networks by the governance structure of the system as a whole. At the same time, new tensions may arise. A good example is the potential tension between a top-down role of the network as a tool of regional administration pursuing regional objectives and the bottom-up role of representing the needs and priorities of participant organizations. Even in this case, different governance modes of the network may exhibit different advantages and disadvantages. Our study also shows that another component of the general institutional environment can play a role in assuring the functionality of networks. Both NAO and lead organization modes work around a structured organization with a specific governance structure. As it defines how different interests are represented in decision-making processes and the overall functioning of the central organization of the network, such a structure may influence the capacity of the network to respond to tension and achieve its goals. In synthesis, there is a link between the governance mode analysis and the regional governance framework on the one hand, and on the other hand, the governance structure of the specific network’s central organization.

In terms of policy implications, the cases show two different interpretations of the role that mandated networks can play in the process of institutional change aiming at a “less decentralized” way of functioning of the regional system. In one case (Tuscany), the network evolved in the direction of becoming a substantial part of the regional government and administration. Here, the critical issue is how to exploit the advantages of a specialized form of government devoted to a certain sector without paying too high a price for coordination with other components of the administrative machine. From this point of view (being part of the “governmental machine”), the positive Tuscany experience confirms that oncological care remains a definite subsystem in the general healthcare system and is a very specific area in terms of the competencies needed in order to be governed. From the other, the Tuscany experience also confirms the role that political cultures and stability play in the governance forms of public healthcare systems.

In Veneto, the network moved in the opposite direction, emphasizing its role as a governing body of professional behaviors. The network was able to benefit from changes in the system in two different ways: a more efficient link and distribution of tasks with the regional government (Azienda Zero in particular) and easier relationships with professionals due to a clearer representation of provider roles (hubs and spokes) in the new design of LHOs boundaries. In this process, the flexibility of the LO form of governance helped.

Both cases showed how the two networks reacted to a change in the institutional environment, but their reactions were for the most part not intentionally led and broadly oriented by their basic nature (more or less institutionalized). Structure and functioning of mandated network should be the result of explicit decisions, in particular the design should take into account the overall institutional framework in which they operate. If the specific context makes spontaneous adjustments not a satisfying or viable option, our study may offer some indications for intentional interventions in terms of possible evolution and condition to be respected.

## Data Availability

The data used in this manuscript are from recorded and transcribed interviews. It is not possible to share the research data publicly, since individual privacy could be compromised. The datasets are available from the corresponding author on reasonable request and with permission from interviewees.

## Appendix

### Interview Protocol

#### Introduction

Our aim is to understand how clinical networks work inside the governance of the regional healthcare system and to analyze and compare the governance of two cancer networks in two Italian regions that underwent system reconfiguration processes.

#### Questions

1. What are the functions and main goals of the network?

2. What is your contribution to network functioning?

3. Do the functions of the network interfere with your responsibilities? Does this have a positive or negative effect? In other words, does the network help you or is it an obstacle to your role?

4. Does the functioning of the network depend on the design of the network itself, on previous and consolidated relationships, or on personal elements?

5. What are the governance mechanisms the regional government has adopted to enhance relationships in the network?

6. Is there an organization that leads the network and what is its role?

7. What are the governance structures of the networks and their roles? Is there a “network broker” figure in the network and what role does he/she have?

8. What are the roles of stakeholders (professionals, LHO/UHO directions, regional authorities)?

9. Who controls resources? Does the network have its own resources? Which resources are controlled centrally by the network and which are controlled by single nodes?

10. Are there tensions between central nodes and peripheral nodes?

11. Do you perceive the network as effective?

12. Have there been changes in network governance after healthcare system reorganization? Which reconfigurations (LHO mergers, intermediate body creation, etc.) had the greatest effect on network governance?

13. Could you give us a synthesis of positive and negative elements of the network?

14. Have there been changes in terms of facilitating relations between professionals, length of procedures, formal governance structures, and responsibilities assigned to the network in regional governance?

## Abbreviations

CEO: Chief executive officer
DRG: Diagnosis-related groups
IOSPN: Institute for Oncologic Studies, Prevention and Networks
LHO: Local health organization
LO: Lead organization
NAO: Network administrative organization
ONC: Oncological network coordination
SC: Scientific committee
STC: Strategic committee
TC: Technical committee
UHO: University hospital organization
VOI: Veneto Oncological Institute

## References

[1] Addicott R, Ferlie E. Understanding power relationships in health care networks. J Health Organ Manag. 2007. 10.1108/14777260710778925. 10.1108/14777260710778925 17933371

[2] Tremblay D, Touati N, Roberge D, Breton M, Roch G, Denis JL, et al. Understanding cancer networks better to implement them more effectively: a mixed methods multi-case study. Implement Sci. 2016. 10.1186/S13012-016-0404-8. 10.1186/s13012-016-0404-8 PMC4802906 27000152

[3] Rodríguez C, Langley A, Béland F, Denis JL. Governance, power, and mandated collaboration in an interorganizational network. Adm Soc. 2007. 10.1177/0095399706297212.

[4] Iedema R, Verma R, Wutzke S, Lyons N, McCaughan B. A network of networks: the governance of deliberative approaches to healthcare improvement and reform. J Health Organ Manag. 2017. 10.1108/JHOM-07-2016-0146. 10.1108/JHOM-07-2016-0146 28482771

[5] Alberti VF, Alberti VF, Tozzi VD, Pinelli N, Sartirana M (2012). Le ragioni e gli obiettivi della ricerca. Il ruolo delle aziende sanitarie nelle reti cliniche in Italia.

[6] Provan KG, Kenis P. Modes of network governance: structure, management, and effectiveness. J Publ Adm Res Theor. 2008. 10.1093/jopart/mum015.

[7] Willem A, Gemmel P. Do governance choices matter in health care networks? An exploratory configuration study of health care networks. BMC Health Serv Res. 2013. 10.1186/1472-6963-13-229. 10.1186/1472-6963-13-229 PMC3727985 23800334

[8] Raab J, Mannak RS, Cambré B. Combining structure, governance, and context: a configurational approach to Network Effectiveness. J Publ Adm Res Theor. 2015. 10.1093/jopart/mut039.

[9] Kenis P, Provan KG. Towards an exogenous theory of public network performance. Public Adm. 2009. 10.1111/j.1467-9299.2009.01775.x.

[10] Popp JK, Casebeer A. Be careful what you ask for: things policy-makers should know before mandating networks. Health Manage Forum. 2015. 10.1177/0840470415599113. 10.1177/0840470415599113 26347480

[11] Saz-Carranza A. The power dynamics of mandated network administrative organizations. Public Adm Rev. 2015. 10.1111/puar.12445.

[12] Ferlie E, Fitzgerald L, McGivern G, Dopson S, Bennett C (2013). Making wicked problems governable? The case of managed networks in health care.

[13] Cantarelli P, Lega F, Longo F. La regione capogruppo sanitaria: assetti istituzionali e modelli organizzativi emergenti. In: Cergas Bocconi, editor. Rapporto OASI 2017. Milano: Egea; 2017. p. 363–80.

[14] Oliver C. Determinants of interorganizational relationships: integration and future directions. Acad Manag Rev. 1990. 10.2307/258156.

[15] Turrini A, Cristofoli D, Frosini F, Nasi G. Networking literature about determinants of network effectiveness. Public Adm. 2010. 10.1111/j.1467-9299.2009.01791.x.

[16] Ouchi WG. Markets, bureaucracies, and clans. Adm Sci Q. 1980. 10.2307/2392231.

[17] Mcguire M, Agranoff R. The limitations of public management networks. Public Adm. 2011. 10.1111/j.1467-9299.2011.01917.x.

[18] Provan KG, Lemaire RH. Core concepts and key ideas for understanding public sector organizational networks: using research to inform scholarship and practice. Public Adm Rev. 2012. 10.1111/j.1540-6210.2012.02595.x.

[19] Uzzi B. Social structure and competition in interfirm networks: the paradox of embeddedness. Adm Sci Q. 1997. 10.2307/2393808.

[20] Segato F, Raab J. Mandated network formation. Int J Public Sect Manag. 2019. 10.1108/IJPSM-01-2018-0018.

[21] Cristofoli D, Markovic J, Meneguzzo M. Governance, management and performance in public networks : how to be successful in shared-governance networks. J Manag Gov. 2014. 10.1007/s10997-012-9237-2.

[22] McInnes E, Haines M, Dominello A, Kalucy D, Jammali-Blasi A, Middleton S, Klineberg E. What are the reasons for clinical network success? A qualitative study. BMC Health Serv Res. 2015. 10.1186/s12913-015-1096-5. 10.1186/s12913-015-1096-5 PMC4635586 26541410

[23] McInnes E, Middleton S, Gardner G, Haines M, Haertsch M, Paul CL, Castaldi P. A qualitative study of stakeholder views of the conditions for and outcomes of successful clinical networks. BMC Health Serv Res. 2012. 10.1186/1472-6963-12-49. 10.1186/1472-6963-12-49 PMC3325167 22373078

[24] Agranoff R, McGuire M, Mandell M (2001). After the network is formed. process, power and performance. Getting results through collaboration networks and network structures for public policy and management.

[25] Ferlie E, Fitzgerald L, McGivern G, Dopson S, Exworthy M (2010). Networks in health care: a comparative study of their management, impact and performance. Report for the National Institute for Health Research Service Delivery and Organisation programme, Queen’s Printer and Controller of HMSO.

[26] Kapucu N, Van Wart M. The evolving role of the public sector in managing catastrophic disasters: lessons learned. Adm Soc. 2006. 10.1177/0095399706289718.

[27] Park SH. Managing an interorganizational network: a framework of the institutional mechanism for network control. Organ Stud. 1996. 10.1177/017084069601700505.

[28] Watts DJ, Strogatz SH. Collective dynamics of “small-world” networks. Nature. 1998. 10.1038/30918. 10.1038/30918 9623998

[29] Dagnino GB, Levanti G, Mocciaro L, Destri A. Structural dynamics and intentional governance in strategic interorganizational network evolution: a multilevel approach. Organ Stud. 2016. 10.1177/0170840615625706.

[30] Gulati R, Sytch M, Tatarynowicz A. The rise and fall of small worlds: exploring the dynamics of social structure. Organ Sci. 2012. 10.1287/orsc.1100.0592.

[31] Sheaff R, Benson L, Farbus L, Schofield J, Mannion R, Reeves D. Network resilience in the face of health system reform. Soc Sci Med. 2010. 10.1016/j.socscimed.2009.11.011. 10.1016/j.socscimed.2009.11.011 20056304

[32] Burt RS (2005). Brokerage and closure: an introduction to social capital.

[33] Uzzi B, Spiro J. Collaboration and creativity: The small world problem. Am J Sociol. 2005. 10.1086/432782.

[34] Eisenhardt KM. Building theories from case study research. Acad Manag Rev. 10.2307/258557.

[35] Yin RK (1999). Enhancing the quality of case studies in health services research. Health Serv Res.

[36] Miles M, Huberman A (1994). Qualitative data analysis.

[37] Morando V, Tozzi V. Processi evolutivi delle reti oncologiche tra dinamiche istituzionali e manageriali. In: Cergas Bocconi, editor. Rapporto OASI 2014. Milano: Egea; 2014. p. 521–51.

[38] Del Vecchio M, Romiti A (2017). Il riaccentramento nel contesto pubblico: implicazioni per il governo dei sistemi e delle aziende sanitarie. Azienda Pubblica.

[39] Prades J, Morando V, Tozzi VD, Verhoeven D, Germà JR, Borras JM. Managing cancer care through service delivery networks: the role of professional collaboration in two European cancer networks. Health Serv Manag Res. 2018. 10.1177/0951484817745219. 10.1177/0951484817745219 29239683

[40] Leavy P (2014). The Oxford handbook of qualitative research.

[41] Boeije H. A purposeful approach to the constant comparative method in the analysis of qualitative interviews. Qual Quant. 2002. 10.1023/A:1020909529486.

[42] Lambotte F, Meunier D. From bricolage to thickness: making the most of the messiness of research narratives. Qual Res Org Manage Int J. 2013. 10.1108/17465641311327531.

[43] Denzin NK, Lincoln YS, Denzin NK, Lincoln YS (2011). Disciplining the practice of qualitative research. The sage handbook of qualitative research.

[44] Jennings G, Kensbock S, Junek O, Radel K, Kachel U. Lived experiences of early career researchers: learning about and doing grounded theory. J Hosp Tour Manag. 2010. 10.1375/jhtm.17.1.21.

[45] Yin RK (2011). Qualitative research from start to finish.

[46] Raich M, Müller J, Abfalter D. Hybrid analysis of textual data. Manag Decis. 2014. 10.1108/MD-03-2012-0247.

[47] Flick U (2009). An introduction to qualitative research.

[48] Del Vecchio M, Romiti A, Del Vecchio M, Pinelli N, Ripa di Meana F, Romiti A, Tanese A (2017). Nuovi Scenari per la governance delle aziende e dei sistemi sanitari regionali. Aziende e management per il futuro del SSN.

[49] Lega F, Tozzi VD, Cantù E (2009). Il cantiere delle reti cliniche in Italia: analisi e confronto di esperienze in oncologia. L’aziendalizzazione della sanità in Italia. Rapporto OASI 2009.

[50] Hillebrand B, Biemans W. The relationship between internal and external cooperation: literature review and propositions. J Bus Res. 2003. 10.1016/S0148-2963(01)00258-2.

